# A Simplified Baseband Prefilter Model with Adaptive Kalman Filter for Ultra-Tight COMPASS/INS Integration

**DOI:** 10.3390/s120709666

**Published:** 2012-07-17

**Authors:** Yong Luo, Wenqi Wu, Ravindra Babu, Kanghua Tang, Bing Luo

**Affiliations:** 1 College of Mechatronics and Automation, National University of Defense Technology, Changsha 410073, China; E-Mails: wenqiwu_lit@hotmail.com (W.W.); tt_kanghua@hotmail.com (K.T.); ruobing@nudt.edu.cn (B.L.); 2 Advance Technology Labs, Wipro Technologies, Chennai, India; E-Mail: s.ravi@unswalumni.com

**Keywords:** COMPASS, ultra-tight COMPASS/INS integration, simplified prefilter model, adaptive Kalman filter

## Abstract

COMPASS is an indigenously developed Chinese global navigation satellite system and will share many features in common with GPS (Global Positioning System). Since the ultra-tight GPS/INS (Inertial Navigation System) integration shows its advantage over independent GPS receivers in many scenarios, the federated ultra-tight COMPASS/INS integration has been investigated in this paper, particularly, by proposing a simplified prefilter model. Compared with a traditional prefilter model, the state space of this simplified system contains only carrier phase, carrier frequency and carrier frequency rate tracking errors. A two-quadrant arctangent discriminator output is used as a measurement. Since the code tracking error related parameters were excluded from the state space of traditional prefilter models, the code/carrier divergence would destroy the carrier tracking process, and therefore an adaptive Kalman filter algorithm tuning process noise covariance matrix based on state correction sequence was incorporated to compensate for the divergence. The federated ultra-tight COMPASS/INS integration was implemented with a hardware COMPASS intermediate frequency (IF), and INS's accelerometers and gyroscopes signal sampling system. Field and simulation test results showed almost similar tracking and navigation performances for both the traditional prefilter model and the proposed system; however, the latter largely decreased the computational load.

## 1. Introduction

The global COMPASS or Beidou II is a second generation Chinese satellite navigation system being developed from its first generation predecessor, Beidou I, which was a regionally-based system [1,2]. By the end of 25 February 2012 eleven COMPASS satellites have been launched successfully. Currently, COMPASS is providing reliable position services to the south and southeast coastland of China and south Asian areas [3].

Like a GPS receiver, the COMPASS receiver also faces the paradoxical situation in optimising the carrier-tracking loop bandwidth to guarantee anti-jamming capability and dynamics adaptation simultaneously, *i.e.*, anti-jamming capability needs a narrow bandwidth while dynamics adaptation needs a wider one [4,5]. Ultra-tight GPS/INS integration was actually proposed to solve this problem [6–11]. The basic concept behind the ultra-tight integration approach is that, the dynamics of the GPS receiver measured by an INS can be integrated with the GPS tracking loop, which results in ‘dynamic-free’ GPS signals [12] that enters the tracking loop, so that GPS receiver's anti-jamming capability and dynamic adaptation can be guaranteed simultaneously. In this paper, the discussion is based on COMPASS B3 frequency signal which share many features in common with the GPS L1 frequency signal [1,2], and therefore the previous discussions on ultra-tight GPS/INS integration are applicable to COMPASS/INS system with minor modifications.

**Figure 1.**
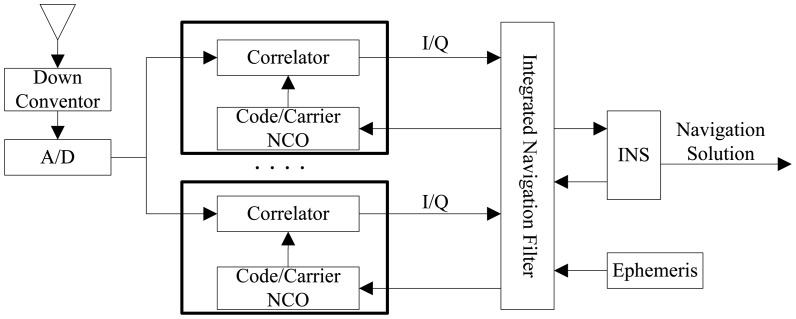
Central design of ultra-tight GPS/INS integration.

Generally, the ultra-tight GPS/INS integrated navigation systems can be classified as central architecture [6,8] and federated architecture [9,10]. In central architecture, carrier and code tracking and INS corrections are performed simultaneously in a single integrated Kalman filter, as shown in Figure 1 [13]. The kernel of its implementation lies in establishing the mathematical relationship between I/Q measurements and INS error states (position, velocity, attitude, gyroscope and accelerator bias errors), which is nonlinear. In addition, if six measurements are contained in each channel, for *N* channels a 6 × *N* dimension measurement information need to be processed. For this reason, the central architecture is difficult to implement in real-time applications, and therefore the discussion primarily focuses on the federated architecture in this paper.

**Figure 2.**
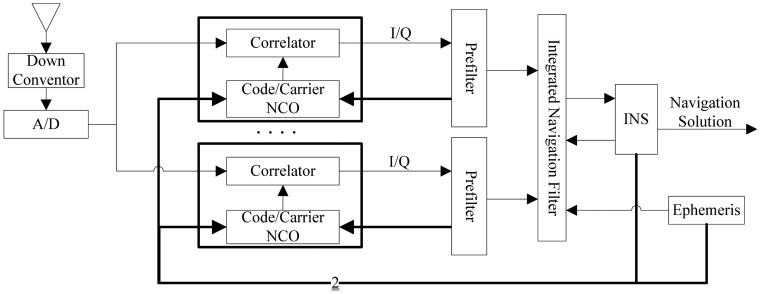
Federated design of ultra-tight GPS/INS integration.

In federated architecture, the large integrated Kalman filter is decomposed into two filters operating at different rates [7], as shown in Figure 2 [13]. The code and carrier tracking loops are completed in a baseband signal pre-processing filter (prefilter for short) in each channel, and the integrated navigation filter (master filter) is used to process the output of the prefilters and restrict the INS errors. Different prefilter models and their impact on GPS signal tracking have been discussed in [14,15]. Generally, the normalized signal amplitude, carrier phase tracking error, carrier frequency tracking error, carrier frequency rate tracking error and code phase tracking error are included in the state space of prefilter, and the code and carrier discriminator outputs are used as measurements to ensure a linear Kalman filter implementation for the prefilter.

A common characteristic of traditional prefilter models is that the signal amplitude is independent of other state variables and discriminator outputs, such that the observability of normalized signal amplitude would be a substantial problem in actual implementation [16]. It is well known that carrier tracking has more stringent requirements than the code tracking [4,5], besides, the code Doppler frequency is proportional to the carrier frequency, and the code phase can be controlled by shifting the code rate [17]. Therefore, the carrier tracking is emphasized during the prefilter implementation.

This paper has proposed a simplified prefilter model and a corresponding adaptive Kalman filter algorithm to replace the traditional one. The state space of this simplified prefilter model consists of only carrier phase tracking error, carrier frequency tracking error and carrier frequency rate tracking error, and the two-quadrant arctangent discriminator output is used as a measurement. Since the code tracking error component has been excluded from the state space, if the code/carrier divergence was ignored it will destroy the carrier tracking process [11,18]. An adaptive Kalman filter algorithm was therefore used to compensate for the code/carrier divergence where the process noise covariance was tuned online based on state correction sequence [19]. The performance of federated ultra-tight COMPASS/INS integration with this simplified adaptive prefilter (S-AKF for short) has been compared with the traditional filter (T-KF for short). Two sets of data, collected in a field environment and with a complex GNSS/INS signal hardware simulator respectively, were used to assess the performance. Test results showed a more or less identical tracking and navigation performance of S-AKF with that of T-KF; however, S-AKF largely reduced the computational load.

The remainder of this paper is organized as follows: Section 2 introduces a commonly used prefilter and integrated navigation filter models in federated GPS/INS architecture, Section 3 introduces the simplified prefilter model and corresponding adaptive Kalman filter algorithm, in addition, the COMPASS/INS integrated navigation filter model is also proposed. Section 4 evaluates the performance of S-AKF and T-KF through simulation and field experiments. The paper finishes with conclusions and an outline of future work in Section 5.

## 2. Traditional Filter Model in Federated GPS/INS Integration

**Figure 3.**
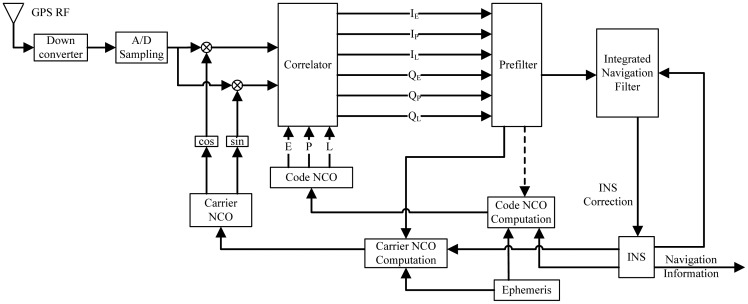
Federated ultra-tight GPS/INS integration with one channel in detail.

Before introducing the simplified adaptive prefilter model of ultra-tight COMPASS/INS integration, a brief introduction is given on traditional prefilter and integrated navigation model of federated GPS/INS integration. Expanding Figure 2 for a single channel, the architecture of federated ultra-tight GPS/INS integration is shown in the Figure 3 [7,11]. Prefilter and integrated navigation filter are two kernel components of this architecture.

### 2.1. Integrated Navigation Filter Model

Traditionally, the state vector of navigation filter is defined as [11]:

(1) $$X={\left[\delta R^{e}\;\delta V^{e}\;\delta \psi^{e}\;\delta \omega^{b}\;\delta A^{b}\;\delta T\right]}^{T}$$

where *δ****R****^e^* is position error, *δ****V****^e^* is velocity error, *δ****ψ****^e^* is attitude error, *δ****ω****^b^* is gyroscope bias error, *δ****A****^b^* is accelerometer bias error, *δ****T*** = [*δb δd*]*^T^* is the clock error vector, the superscript *e* and *b* represent ECEF frame and body frame respectively.

The corresponding system model of navigation filter is:

(2) $$\left[\begin{array}{c} \delta {\dot{R}}^{e}\\ \delta {\dot{V}}^{e}\\ \delta {\dot{\psi }}^{e}\\ \delta {\dot{\omega }}^{b}\\ \delta {\dot{A}}^{b}\\ \delta T \end{array}\right]=\left[\begin{array}{cccccc} 0 & I & 0 & 0 & 0 & 0\\ G-{\tilde{\Omega }}^{e}{\tilde{\Omega }}^{e} & -2{\tilde{\Omega }}^{e} & {\tilde{A}}^{e} & 0 & C_{b}^{e} & 0\\ 0 & 0 & -{\tilde{\Omega }}^{e} & -C_{b}^{e} & 0 & 0\\ 0 & 0 & 0 & 0 & 0 & 0\\ 0 & 0 & 0 & 0 & 0 & 0\\ 0 & 0 & 0 & 0 & 0 & \Lambda \end{array}\right]\;\left[\begin{array}{c} \delta R^{e}\\ \delta V^{e}\\ \delta \psi^{e}\\ \delta \omega^{b}\\ \delta A^{b}\\ \delta T \end{array}\right]+\left[\begin{array}{c} w_{1}\\ w_{2}\\ w_{3}\\ w_{4}\\ w_{5}\\ w_{6} \end{array}\right]$$

$$\text{where}\;{\tilde{A}}^{e}=\left[\begin{array}{ccc} 0 & f_{z} & -f_{y}\\ -f_{z} & 0 & f_{x}\\ f_{y} & -f_{x} & 0 \end{array}\right],{\tilde{\Omega }}^{e}=\left[\begin{array}{ccc} 0 & \omega_{e} & 0\\ -\omega_{e} & 0 & 0\\ 0 & 0 & 0 \end{array}\right],\Lambda =\left[\begin{array}{cc} 0 & 1\\ 0 & 0 \end{array}\right],G=\frac{\mu }{r^{3}}\left[\begin{array}{ccc} -1+\frac{3x^{2}}{r^{2}} & \frac{3xy}{r^{2}} & \frac{3xz}{r^{2}}\\ \frac{3xy}{r^{2}} & -1+\frac{3y^{2}}{r^{2}} & \frac{3yz}{r^{2}}\\ \frac{3xz}{r^{2}} & \frac{3yz}{r^{2}} & -1+\frac{3z^{2}}{r^{2}} \end{array}\right]$$

where [*x y z*] is the vehicle's current position in ECEF-frame, $r=\sqrt{x^{2}+y^{2}+z^{2}}[f_{x}f_{y}f_{z}]$, is the specific force vector in the body frame, $C_{b}^{e}$ is the body-to-Earth-frame coordinate transformation matrix, and ***ω****_e_* is the Earth's angular rate [20].

For the integrated navigation filter, the error components of pseudo range, pseudo range rate and pseudo range acceleration are used as measurements which are proportional to corresponding estimated states of prefilter [11]. Since the emphasis was on the prefilter model, the measurement equation was deliberately omitted here, for more details please refer to [11,21].

### 2.2. Traditional Prefilter Model

In federated ultra-tight GPS/INS integration, the prefilters are responsible for implementing code and carrier tracking, and also providing measurement information and corresponding measurement noise matrices for the integrated navigation filter [9–11]. For specific appliactions different prefilter models have been investigated [14,15]. A most commonly used prefilter model will be introduced here, the performance of which will be compared with that of a simplified prefilter model with an adaptive Kalman filter. The system model for the prefilter is written as follows [14]:

(3) $$\left[\begin{array}{c} \dot{A}\\ \delta \dot{\tau }\\ \delta \dot{\phi }\\ \delta \dot{f}\\ \delta \dot{a} \end{array}\right]=\left[\begin{array}{ccccc} 0 & 0 & 0 & 0 & 0\\ 0 & 0 & 0 & \frac{\lambda_{L1}}{2\pi \lambda_{CA}^{L1}} & 0\\ 0 & 0 & 0 & 1 & 0\\ 0 & 0 & 0 & 0 & 1\\ 0 & 0 & 0 & 0 & 0 \end{array}\right]\;\left[\begin{array}{c} A\\ \delta \tau \\ \delta \phi \\ \delta f\\ \delta a \end{array}\right]+\left[\begin{array}{ccccc} 1 & 0 & 0 & 0 & 0\\ 0 & 1 & \frac{\lambda_{L1}}{2\pi \lambda_{CA}} & 0 & 0\\ 0 & 0 & 1 & 0 & 0\\ 0 & 0 & 0 & 1 & 0\\ 0 & 0 & 0 & 0 & 1 \end{array}\right]\;\left[\begin{array}{c} w_{A}\\ w_{\tau }\\ w_{\phi }\\ w_{f}\\ w_{a} \end{array}\right]$$

where *A* is the normalized signal amplitude, *δØ* is the carrier phase tracking error, *δf* is the carrier frequency error, *δa* is the carrier frequency rate error, *δτ* is the code phase error, *λ*_L1_ ≈ 0.19 *m* is the GPS L1 carrier wavelength, $\lambda_{CA}^{L_{1}}\approx 293m$ is the GPS *CA* code chip length, *w_A_* is the process noise for the normalized signal amplitude, *w_τ_* represents the code/carrier divergence, *w_Ø_* represents the carrier phase noise due to the clock bias, *w_f_* represents the carrier frequency noise due to the clock drift, and *w_a_* represents the carrier phase acceleration noise due to the receiver dynamics.

The outputs of normalized early-minus-late envelope code discriminator and two-quadrant arctangent carrier discriminator are used as measurements, and the measurement equation is written as follows [14,15]:

(4) $$Z=\left[\begin{array}{c} \delta \tau \\ \delta \phi \end{array}\right]=\left[\begin{array}{ccccc} 0 & 1 & 0 & 0 & 0\\ 0 & 0 & 1 & 0 & 0 \end{array}\right]\;\left[\begin{array}{c} A\\ \delta \tau \\ \delta \phi \\ \delta f\\ \delta a \end{array}\right]=\left[\begin{array}{c} \frac{\sqrt{I_{E}^{2}+Q_{E}^{2}}-\sqrt{I_{L}^{2}+Q_{L}^{2}}}{\sqrt{I_{E}^{2}+Q_{E}^{2}}+\sqrt{I_{L}^{2}+Q_{L}^{2}}}\\ \tan^{-1}(Q_{P}/I_{P}) \end{array}\right]+\left[\begin{array}{c} \nu_{1}\\ \nu_{2} \end{array}\right]$$

where *v*_1_ and *v*_2_ are the output noises of code and carrier discriminators respectively.

As shown in Equation (3), the code phase error *δτ* is proportional to the carrier frequency error *δf*, and in order to decrease the state space dimension the “carrier aided code tracking process” can be implemented “outside” the prefilter. In addition, as shown in Equation (4), if the normalized discriminators were used the measurements would contain no information of estimated signal amplitude, and the observability of which would be a substantial problem [16]. In some cases, the baseband I/Q information were used as measurements directly [14,15] including the signal amplitude; however, a non-linear filter algorithm is required to implement code and carrier tracking [7,14,15]. Considering the above analysis, the code phase error and signal amplitude will be excluded from the state space of prefilter in the following discussion.

## 3. The Simplified Prefilter Model with Application to Federated Ultra-Tight COMPASS/INS Integration Implementation

A simplified prefilter model for the federated ultra-tight COMPASS/INS integration is investigated for the reduction in the calculation load, and the corresponding integrated navigation filter model is also analyzed.

### 3.1. Simplified Prefilter Model with Adaptive Kalman Filter

For the *jth* (*j* = 1, 2, …, *N*) channel, the system model of the simplified pre-filter is defined as follows (discrete form):

(5) $$X_{k+1}^{j}=\left[\begin{array}{c} \delta \phi_{k+1}^{j}\\ \delta f_{k+1}^{j}\\ \delta a_{k+1}^{j} \end{array}\right]=\left[\begin{array}{ccc} 1 & T & 0.5T^{2}\\ 0 & 1 & T\\ 0 & 0 & 1 \end{array}\right]\;\left[\begin{array}{c} \delta \phi_{k}^{j}\\ \delta f_{k}^{j}\\ \delta a_{k}^{j} \end{array}\right]+\left[\begin{array}{c} w_{\phi }\\ w_{f}\\ w_{a} \end{array}\right]$$

where $\delta \emptyset_{k}^{j}$ (rad) is the carrier phase tracking error at time instant *k*, $\delta f_{k}^{j}$ (rad/s) is the carrier frequency error at time instant *k*, and $\delta a_{k}^{j}$ (rad/s^2^) is the carrier frequency rate error at time instant *k*.

The measurement is the output of two-quadrant arctangent carrier discriminator, and the corresponding measurement model is:

(6) $$Z_{k}^{j}=\tan^{-1}(Q_{p}/I_{p})=[\begin{array}{ccc} 1 & 0 & 0 \end{array}]X_{k}^{j}+\nu_{2}$$

Since the navigation solution accuracy is insufficient for carrier phase tracking [14], the carrier phase is modified by channel filter directly, as shown in Figure 3. The carrier NCO control information is provided as follows:

(7) $$\{\begin{array}{l} {\tilde{\varphi }}_{j,k+1}^{-}=\text{rem}({\tilde{\varphi }}_{j,k}^{+}+(f_{IF}+\Delta {\tilde{f}}_{j,k})\cdot T,2\pi )\\ {\tilde{\varphi }}_{j,k+1}^{+}=\text{rem}({\tilde{\varphi }}_{j,k+1}^{-}X_{k}^{j}(1)+X_{k}^{j}(2)\cdot T+0.5\cdot X_{k}^{j}(3)\cdot T^{2},2\pi ) \end{array}$$

where *f_IF_* represents the centre frequency of down-converted intermediate frequency(IF) COMPASS signal, Δ*f̃_j,k_* represents the carrier Doppler frequency, ${\tilde{\varphi }}_{j,k}^{+}$ and ${\tilde{\varphi }}_{j,k+1}^{+}$ are the carrier phases after prefilter update, ${\tilde{\varphi }}_{j,k}^{-}$ and ${\tilde{\varphi }}_{j,k+1}^{-}$ are the carrier phases before prefilter update, $X_{k}^{j}$ represents the updated state of prefilter, the subscript *k* represents the current time instant, *rem* represents remainder after division operation, and *T* is the prefilter update period.

In Equation (7), the carrier Doppler frequency is obtained from corrected INS information and COMPASS ephemeris as follows:

(8) $$\Delta {\tilde{f}}_{j,k}=\frac{f_{B3}}{c}\langle {\text{LOS}}_{j,k},V_{j,k}^{\text{sat}}-V_{k}^{\text{usr}}\rangle$$

where *f_B_*_3_ is the carrier frequency [1,2], *c* is light velocity, ***LOS****_j,k_* is the unit line-of-sight vector from user to satellite *j*, $V_{j,k}^{\text{sat}}$ and ${\text{V}}_{k}^{\text{usr}}$ represent the satellite and user velocity in ECEF frame respectively, and <,> represents vector dot operation.

Since the code tracking related parameter has been excluded from the state space of this simplified prefilter model, the code tracking is controlled by the carrier tracking process. The code NCO control information is provided as:

(9) $$\{\begin{array}{l} f_{j,k+1}^{\text{code}}=f_{CA}^{B3}-\Delta {\tilde{f}}_{j,k}\frac{f_{CA}^{B3}}{f_{B3}}\\ \Delta \tau_{j,k}=\frac{f_{\text{code},k}^{j}}{f_{\text{sample}}}\\ n_{j,k}=\left[\frac{l_{\text{code}}}{\Delta \tau_{j,k}}\right]\\ {\tilde{\tau }}_{j,k+1}={\tilde{\tau }}_{j,k}+n_{j,k}\cdot \Delta \tau_{j,k}-l_{\text{code}} \end{array}$$

where $f_{CA}^{B3}$ is the code frequency [1,2], *f_sample_* is the sampling frequency, *l_code_* is the code length [1,2], and [***A***] represents the nearest integer greater than or equal to *A*.

Equation (8) shows that the carrier Doppler frequency is obtained from INS information directly and Equation (9) shows that code phase is controlled by code frequency and propagated forward sequentially. As the code frequency is obtained from carrier frequency Doppler, the feedback from traditional prefilter to code NCO computation is omitted as shown in Figure 3 with a dashed arrow.

Comparing Equations (3) and (5), it can be observed that the code/carrier divergence has been excluded from process noises in the simplified prefilter model resulting in degradation in the carrier tracking process [11,18]. Therefore, an adaptive Kalman filter was incorporated to compensate for the code/carrier divergence. Multi-model-based and innovation-based adaptive estimations are most commonly used adaptive Kalman filtering algorithms [19]. Since the simplified prefilter model is already determined an innovation-based adaptive estimation was adopted here. In innovation-based adaptive estimation, the process and measurement noise covariance matrices are adapted using innovation sequences; however, since the code/carrier divergence is a process noise the adaptation emphasis is on $Q_{k}^{j}$ in this manuscript. The initial value of $Q_{k}^{j}$ is calculated as follows:

(10) $${\hat{Q}}_{0}^{j}=\left[\begin{array}{ccc} 2\pi^{2}f_{B3}^{2}\cdot h_{0} & 0 & 0\\ 0 & 8\pi^{4}f_{B3}^{2}\cdot h_{-2} & 0\\ 0 & 0 & (4\pi^{2}f_{B3}^{2}/c^{2})\cdot dR^{2}/dt^{2} \end{array}\right]$$

where *dR*^2^/*dt*^2^ is the vehicle's light of sight acceleration, and *h*_0_ and *h*_−2_ represent the white component and random walk of the oscillator frequency noise [22].

The state correction sequence is used to adapt the process noise covariance matrix $Q_{k}^{j}$ which is computed as follows [19]:

(11) $${\hat{Q}}_{k}^{j}=\frac{1}{N}\sum_{m=m_{0}}^{k}\Delta X_{m}^{j}{\left(\Delta X_{m}^{j}\right)}^{T}+P_{k/k}-\Phi P_{k-1/k-1}\Phi^{T}$$

where $\Delta X_{k}^{j}$ is the state correction sequence which is computed as:

(12) $$\Delta X_{m}^{j}=\Delta X_{m/m}^{j}-\Delta X_{m/m-1}^{j}$$

While a standard Kalman filter, shown in Equation (13), is used to estimate the states of a traditional prefilter model, an adaptive Kalman filter is used for the simplified prefilter model:

(13) $$\{\begin{array}{l} X_{k|k-1}=\Phi X_{k-1|k-1}\\ {\text{P}}_{k|k-1}=\Phi P_{k-1|k-1}\Phi^{T}+{\hat{Q}}_{k-1}\\ K_{k}=P_{k|k-1}H^{T}{\left(HP_{k|k-1}H^{T}+R\right)}^{-1}\\ X_{k|k}=X_{k|k-1}+K_{k}(Z_{k}-HX_{k|k-1})\\ P_{k/k}=(I-K_{k}H)P_{k/k-1} \end{array}$$

Associated matrix multiplication operations in above Kalman filter algorithms are implemented to compare the computational complexities of both filter models. Table 1 shows the number of multiplication operations in detail.

In Table 1, *N* is the window length as used in Equation (11). For example, if *N* = 5 was considered, a $1-\frac{128+45}{503}\approx 65.6\%$ reduction in total number of multiplications is achieved.

### 3.2. Integrated Navigation Filter

The COMPASS B3 frequency signal considered in this paper is BPSK modulated as GPS L1 frequency signal [1,2], the system model of navigation filter is almost in accordance with Equation (2) by just replacing *λ_L_*_1_ with *λ_B_*_3_ and $\lambda_{CA}^{L1}$ with $\lambda_{CA}^{B3}$. However, as in [11,23], only the delta pseudorange and delta pseudorange residuals are chosen as measurements to the navigation filter. For the *jth* channel, the relationship between measurements and estimated states of pre-filter is as follows:

(14) $$\left[\begin{array}{c} \Delta \delta \rho_{k}^{j}\\ \Delta \delta {\dot{\rho }}_{k}^{j} \end{array}\right]=\left[\begin{array}{c} \frac{\lambda_{B3}\cdot (\delta \phi_{k}^{j}-\delta \phi_{k-1}^{j})}{2\pi }\\ \frac{\lambda_{B3}\cdot (\delta f_{k}^{j}-\delta f_{k-1}^{j})}{2\pi } \end{array}\right]=\left[\begin{array}{c} \frac{\lambda_{B3}\cdot (X_{k}^{j}(1)-X_{k-1}^{j}(1))}{2\pi }\\ \frac{\lambda_{B3}\cdot (X_{k}^{j}(2)-X_{k-1}^{j}(2))}{2\pi } \end{array}\right]$$

where *λ_B_*_3_ is the COMPASS B3 carrier wavelength, $X_{k}^{j}$ and $X_{k-1}^{j}$ are the estimated state of prefilter at time instant “*k*” and “*k* − 1”.

The corresponding measurement equation of integrated navigation filter is as follows (only a single channel is list):

(15) $$\left[\begin{array}{c} \Delta \delta \rho_{k}^{j}\\ \Delta \delta {\dot{\rho }}_{k}^{j} \end{array}\right]=\left[\begin{array}{ccccccc} -\Delta {\text{u}}^{\text{T}} & -Tu_{\text{pre}}^{\text{T}} & \frac{T^{2}}{2}u_{\text{pre}}^{\text{T}}{\tilde{A}}^{e} & 0 & \frac{T^{2}}{2}u_{\text{pre}}^{\text{T}}C_{b}^{e} & 0 & T\\ 0 & -\Delta u^{\text{T}} & -Tu_{\text{pre}}^{\text{T}}{\tilde{A}}^{e} & 0 & -Tu_{\text{pre}}^{\text{T}}C_{b}^{e} & 0 & 0 \end{array}\right]\;\left[\begin{array}{c} \delta R^{e}\\ \delta V^{e}\\ \delta \psi^{e}\\ \delta \omega^{b}\\ \delta A^{b}\\ \delta b\\ \delta d \end{array}\right]+\left[\begin{array}{c} \nu_{\Delta \delta \rho_{k}^{j}}\\ \nu_{\Delta \delta {\dot{\rho }}_{k}^{j}} \end{array}\right]$$

where **u***_pre_* is the unit LOS vector from receiver to satellite in previous filter update period, **u** is the unit LOS vector from receiver to satellite in current filter update period, Δ**u** is the difference of above two LOS vectors.

The variances of measurement noises can be obtained from estimation error covariance matrices of pre-filters as follows [11]:

(16) $$R_{k}^{j}=\left[\begin{array}{cc} Var\left(\nu_{\Delta \delta \rho_{k}^{j}}\right) & 0\\ 0 & Var\left(\nu_{\Delta \delta {\dot{\rho }}_{k}^{j}}\right) \end{array}\right]=\left[\begin{array}{cc} {\left(\frac{\lambda_{B3}}{2\pi }\right)}^{2}\left(\begin{array}{l} {\text{P}}_{k}^{j}(3,3)-{\text{P}}_{k-1}^{j}(3,3)-\\ 2T\cdot P_{k-1}^{j}(2,3)-T^{2}\cdot P_{k-1}^{j}(1,3) \end{array}\right) & 0\\ 0 & {\left(\frac{\lambda_{B3}}{2\pi }\right)}^{2}\left(\begin{array}{l} P_{k}^{j}(2,2)-P_{k-1}^{j}(2,2)\\ -2T\cdot P_{k-1}^{j}(1,2) \end{array}\right) \end{array}\right]$$

where $P_{k}^{j}$ and $P_{k-1}^{j}$ are the estimation error covariance matrix of *jth* prefilter at time instant “*k*” and “*k* − 1”.

**Figure 4.**
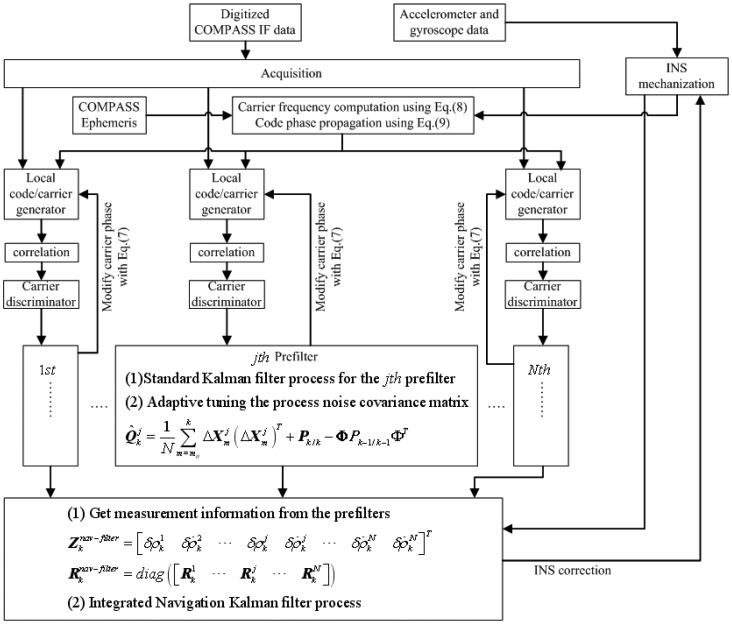
Flow diagram for federated COMPASS/INS integration complementation with the simplified prefilter model and adaptive Kalman filter.

With the simplified prefilter model and adaptive Kalman filter, the federated COMPASS/INS integration implementation process is shown in Figure 4, where **Φ** represents the system matrix of *jth* prefilter defined in Equation (5), $R_{\text{prefilter}}^{j}$ represents the measurement noise variance of *jth* prefilter, $Z_{k}^{\text{nav}-\text{filter}}$ represents the measurements of integrated navigation filter at current instant *k*, and $R_{k}^{\text{nav}-\text{filter}}$ represents the measurement noise covariance of integrated navigation filter at current instant *k*. The flow diagram consists of the following steps:

1. COMPASS IF data was sampled through a hardware sampling system, which will be discussed in “Test description” in detail.
2. The acquisition process gets the initial code phase and carrier Doppler frequency for different visible satellites, which will be used to set the initial values for prefilters.
3. Adaptive Kalman filter algorithm is implemented in each prefilter to implement code and carrier tracking, and provide measurement information to the integrated navigation filter.
4. With measurement information from the prefilters and INS, a classical Kalman filter algorithm is implemented for integrated navigation filter, and the corresponding INS correction information is fed back to update INS errors.
5. Modified INS information and COMPASS ephemeris are used to generate carrier frequency for different satellites [21].
6. Repeat steps (3) to (5).

## 4. Test Description

Federated COMPASS/INS integration with S-AKF and T-KF were implemented in software. Two sets of data were used to compare the performance of S-AKF and T-KF. First, data were collected using a hardware complex GNSS/INS signal simulator to assess the performance in high dynamic case. Second, field data were collected with a COMPASS B3 frequency antenna and an INS to assess the tracking performance of the above two methods.

The comparison of the performance of S-AKF and T-KF is made in both the tracking domain and navigation domain. In tracking domain, Phase Lock Indicator (PLI) and Doppler frequency tracking error are used to evaluate the carrier phase tracking ability. In navigation domain, the position and velocity errors in Earth Centered Earth Fixed (ECEF) frame were compared.

### 4.1. Simulation Test Results

**Figure 5.**
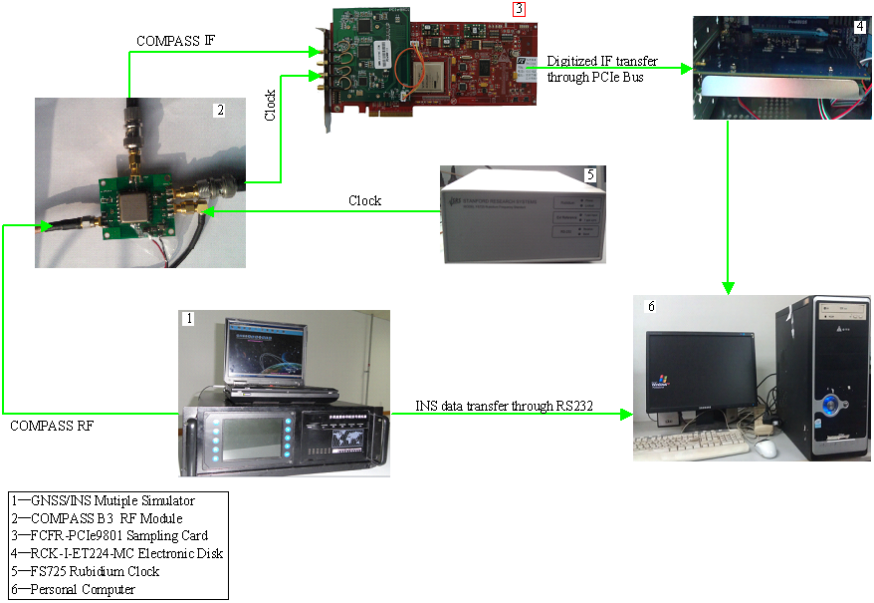
COMPASS IF data and INS data collection process with GNSS/INS hardware simulator.

For the simulation tests, a complex GNSS/INS signal hardware simulator was used to generate the COMPASS radio frequency(RF) signal and INS's accelerometers and gyroscopes data (INS data for short). A hardware sampling system was constructed to sample and store the digitized COMPASS IF signal and INS data. Data collection process for the simulation case is shown in Figure 5.

The data collection system consists of complex GNSS/INS signal hardware simulator, COMPASS B3 RF module, FCFR-PCIe9801 data sampling card [24], RCK-I-ET224-MC electronic disk [25] and FS725 rubidium clock [26]. The function of each component is as follows:

1. GNSS/INS hardware simulator provides synchronized COMPASS B3 frequency RF signal and INS data; the vehicle scenario and signal strength can be configured by users for their corresponding applications.
2. COMPASS B3 RF module is responsible for down-converting B3 RF signal into IF signal and providing driving clock for FCFR-PCIe9801 data sampling card. A reference sampling clock from Rubidium Oscillator is used for the data sampling card.
3. FCFR-PCIe9801 data sampling card completes the data sampling process of IF signal and transfers the sampled data to electronic disk in real time.
4. RCK-I-ET224-MC electronic disk is responsible for storing sampled IF data from sampling card.
5. FS725 rubidium clock provides reference clock for radio frequency module.

The ultra-tight COMPASS/INS integration algorithm was implemented in MATLAB, and the parameters defined in baseband signal processing part are listed in Table 2.

**Figure 6.**
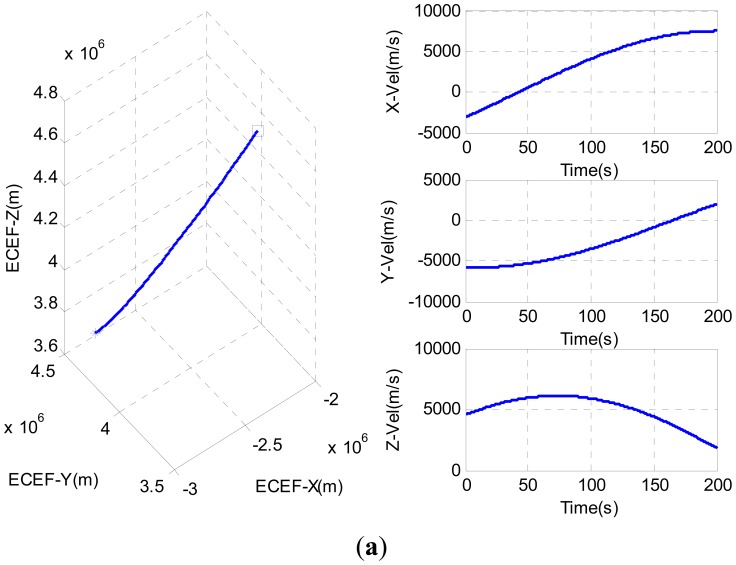

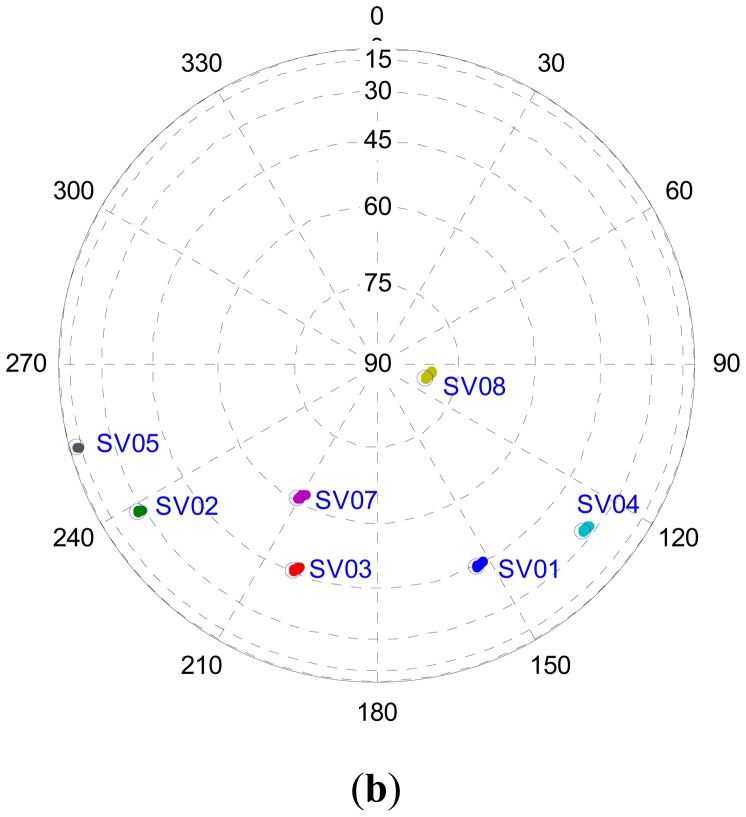
(**a**) The reference trajectory for simulation; (**b**) COMPSS satellite sky-plot in simulation test.

In Table 2, *h*_0_ and *h*_−2_ are quantitative description of oscillator biases of COMPASS B3 RF module and FS725 rubidium clock. With the complex GNSS/INS signal hardware simulator, a reference trajectory with known dynamics was generated with 10 mg accelerometer bias errors and 200 deg/h gyroscope bias errors. The reference position and velocity in ECEF frame are shown in Figure 6(a), where “star” represents the initial position while ‘square’ represents the end. The COMPASS signal strength was kept at −130 dBm (*C*/*N*_0_ ≈ 35 dB − Hz). The satellites 01, 02, 03, 04, 07 and 08 were visible during the simulation test, and the sky plot of the satellites is shown in Figure 6(b).

#### 4.1.1. Tracking Domain Analysis

Phase lock indicator (PLI) and Doppler frequency tracking errors were used to evaluate the tracking performance. The PLI is calculated as described by [4]:

(12) $$PLI_{k}^{j}=\frac{{\left(\sum_{i=1}^{M}I_{P_{i}}^{j}\right)}_{k}^{2}-{\left(\sum_{i=1}^{M}Q_{P_{i}}^{j}\right)}_{k}^{2}}{{\left(\sum_{i=1}^{M}I_{P_{i}}^{j}\right)}_{k}^{2}+{\left(\sum_{i=1}^{M}Q_{P_{i}}^{j}\right)}_{k}^{2}}$$

where $I_{P_{i}}^{j}$ is the in-phase prompt correlator output, $Q_{P_{i}}^{j}$ is the quadra-phase prompt correlator output, and *M* = 20 is the window length chosen for calculating PLI.

Simplifying Equation (12) yields:

(13) $$PLI_{k}^{j}\approx \cos\left(2\delta \phi_{k}^{j}\right)$$

where $\delta \emptyset_{k}^{j}$ is the carrier phase tracking error of *jth* prefilter.

As shown in Equation (13), the value of PLI lies between +1 and −1 and a value of positive one indicates perfect phase lock.

**Figure 7.**
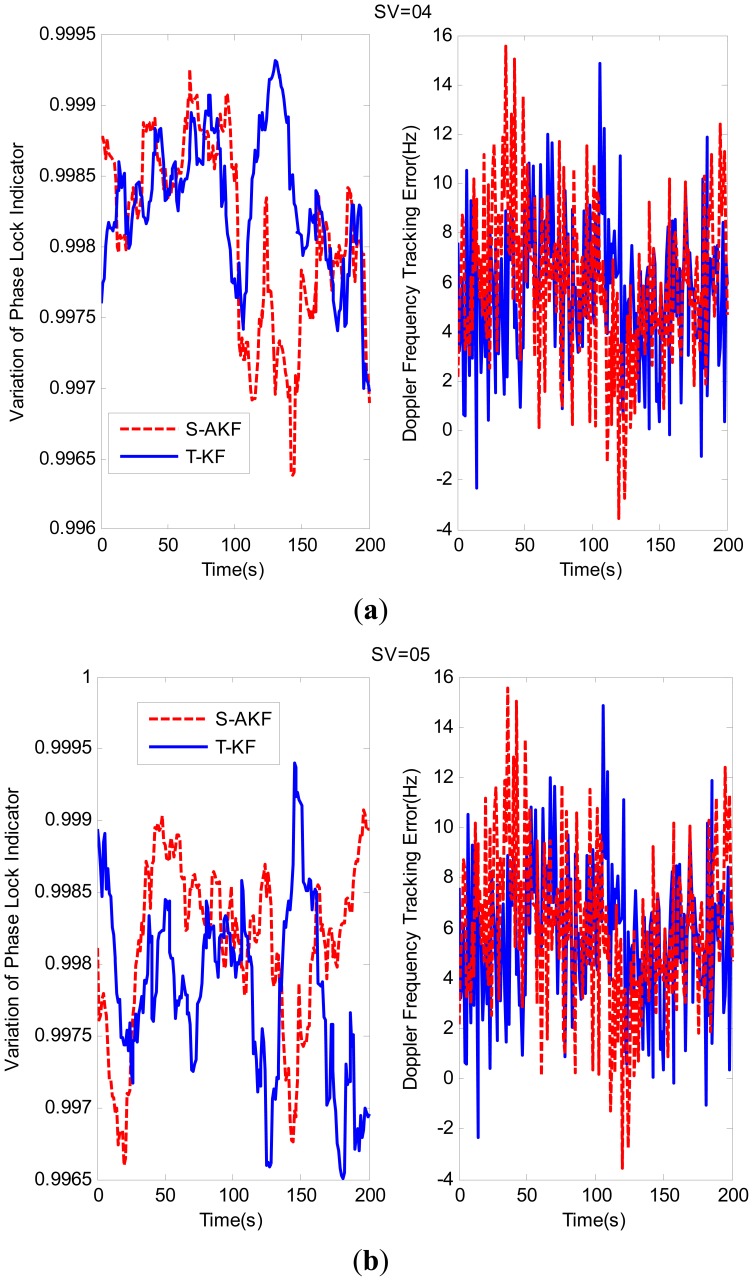
(**a**) Tracking performance comparison for SV04; (**b**) Tracking performance comparison for SV05.

The variations of PLI and Doppler frequency tracking errors for SV04 and SV05 are shown in Figure 7(a,b), respectively. From these figures, the PLI values of S-AKF and T-KF are closer to +1, which indicated that both S-AKF and T-KF are in a near perfect tracking status in this case. The root mean square (RMS) of the tracking Doppler frequency errors are summarized in Table 3 where the S-AKF and T-KF showed an almost similar Doppler frequency tracking errors.

#### 4.1.2. Navigation Domain Analysis

**Figure 8.**
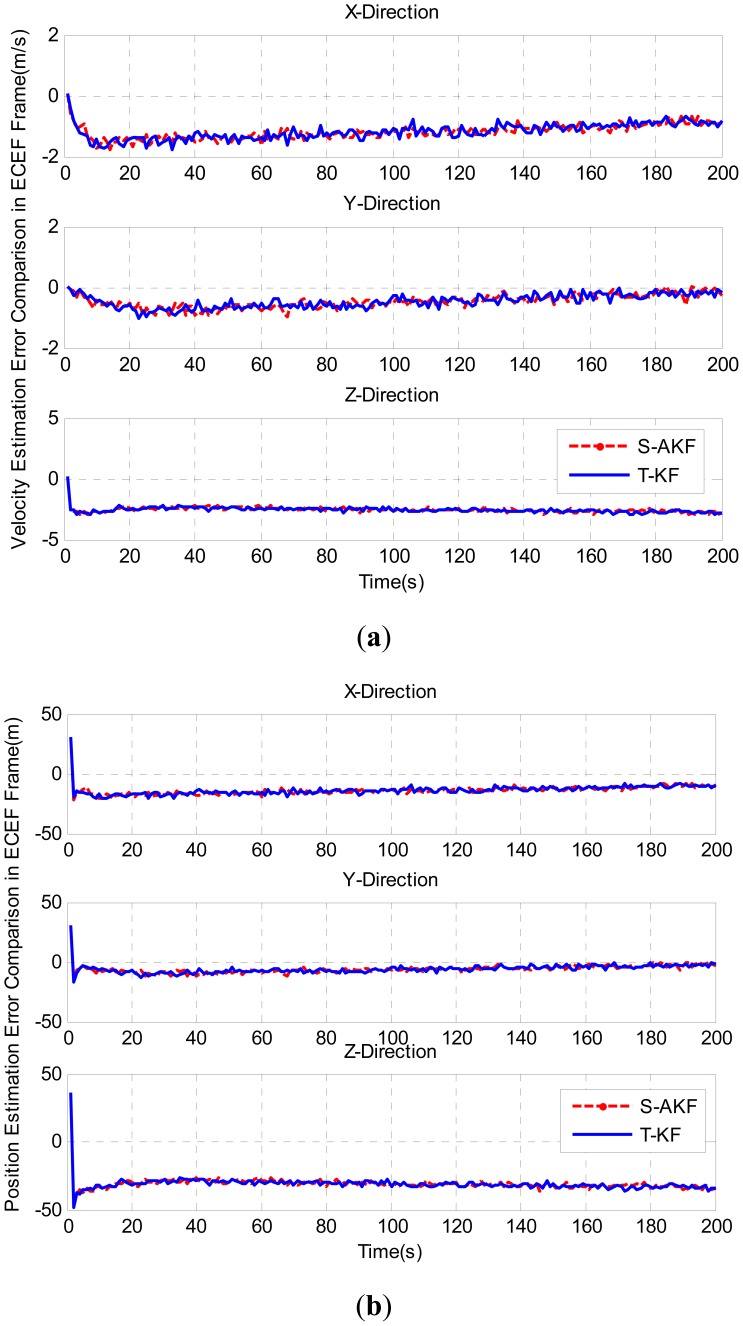
(**a**) Velocity estimation errors of S-AKF and T-KF in ECEF frame; (**b**) Position estimation errors of S-AKF and T-KF in ECEF frame.

For federated COMPASS/INS integrated navigation system with S-AKF and T-KF, the estimated velocity and position errors in ECEF frame were used to compare the navigation performance as shown in Figure 8(a,b), respectively.

The statistics of position and velocity estimation errors are summarized in Table 4 where the S-AKF and T-KF showed very closer velocity and position estimation errors.

### 4.2. Field Test Results

**Figure 9.**
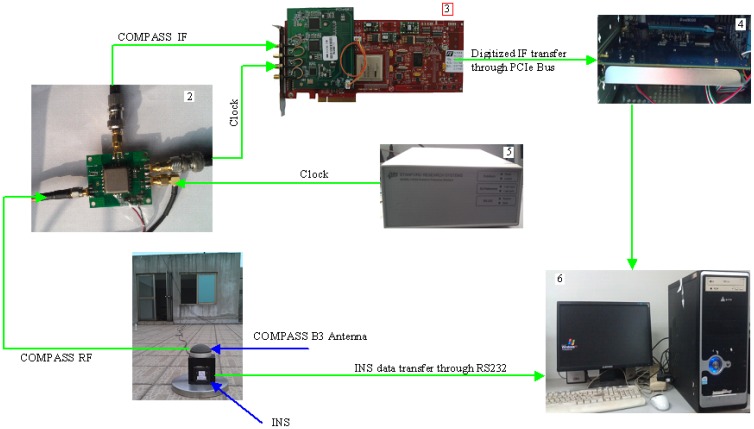
COMPASS IF data and IMU data collection process in field environment.

A field test was conducted to collect real COMPASS B3 frequency IF data and INS data. A COMPASS B3 frequency antenna and an INS were used to replace the complex GNSS/INS signal hardware simulator in simulation case. The corresponding data collection process in the field is shown in the Figure 9. The INS used in this case had 5 mg accelerometer bias errors and 150 deg/h gyroscope bias errors.

**Figure 10.**
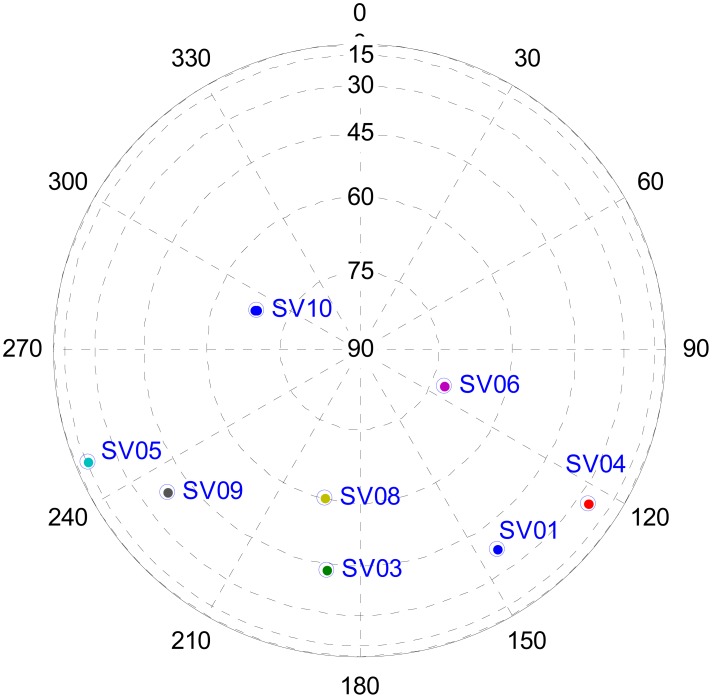
COMPASS satellite sky-plot of field test.

The antenna was located on the roof of an office building to guarantee a strong COMPASS signal (*C*/*N*_0_ ≈ 45 dB − Hz), and the INS was collocated with the antenna. A high precision GPS receiver was used to provide the truth reference of the antenna which are −2,207,210.269, 5,171,488.332 and 3,000,859.525 m in the ECEF frame. The satellites 01, 03, 04, 05, 06, 08, 09 and 10 were visible during the field test, and the sky plot of the satellites is shown in Figure 10.

#### 4.2.1. Tracking Domain Analysis

**Figure 11.**
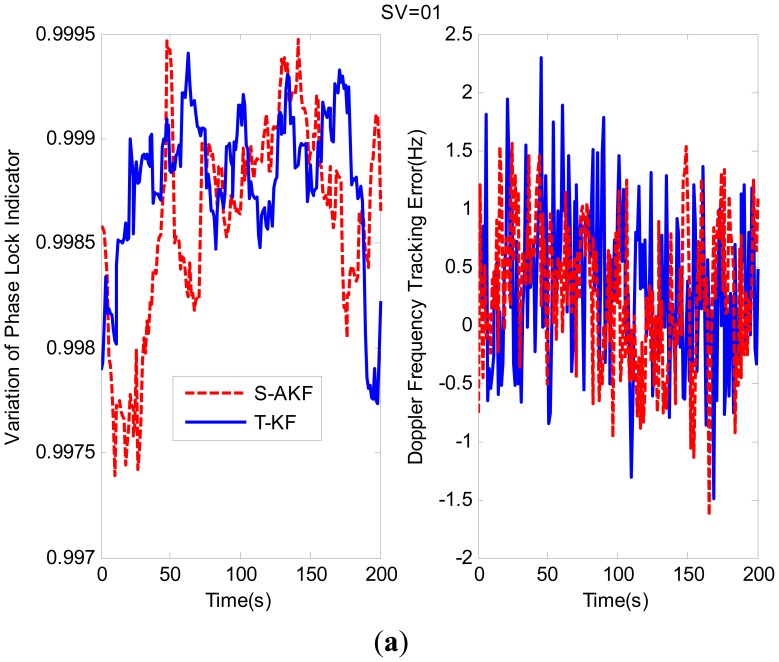

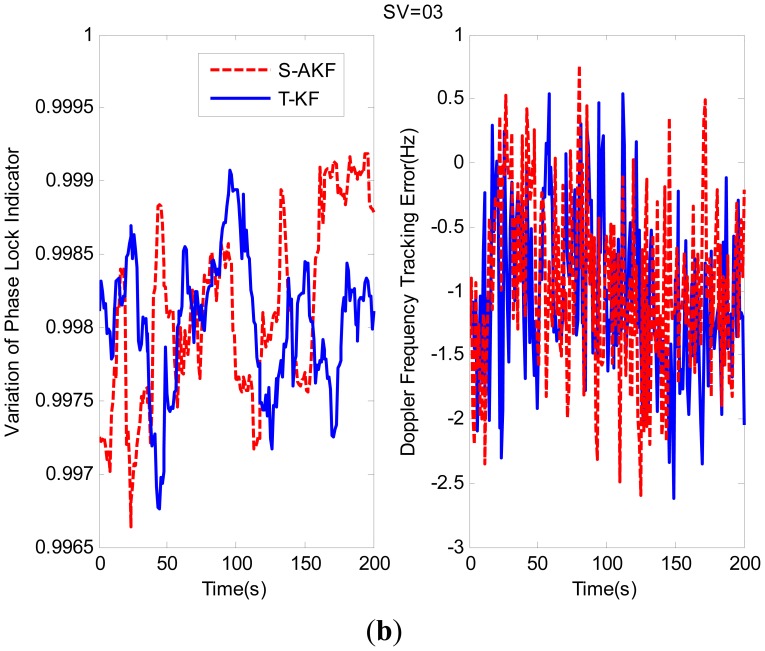
(**a**) Tracking performance comparison for SV01; (**b**) Tracking performance comparison for SV03.

The variations of PLI and Doppler frequency tracking errors for SV01 and SV03 are shown in Figure 11(a,b) respectively. From these figures it can be observed that the PLI values of S-AKF and T-KF are closer to +1 which indicate that both S-AKF and T-KF were in a near perfect tracking status.

The RMS of the tracking Doppler frequency errors are summarized in Table 5. The S-AKF and T-KF showed almost similar Doppler frequency tracking errors.

#### 4.2.2. Navigation Domain Analysis

**Figure 12.**
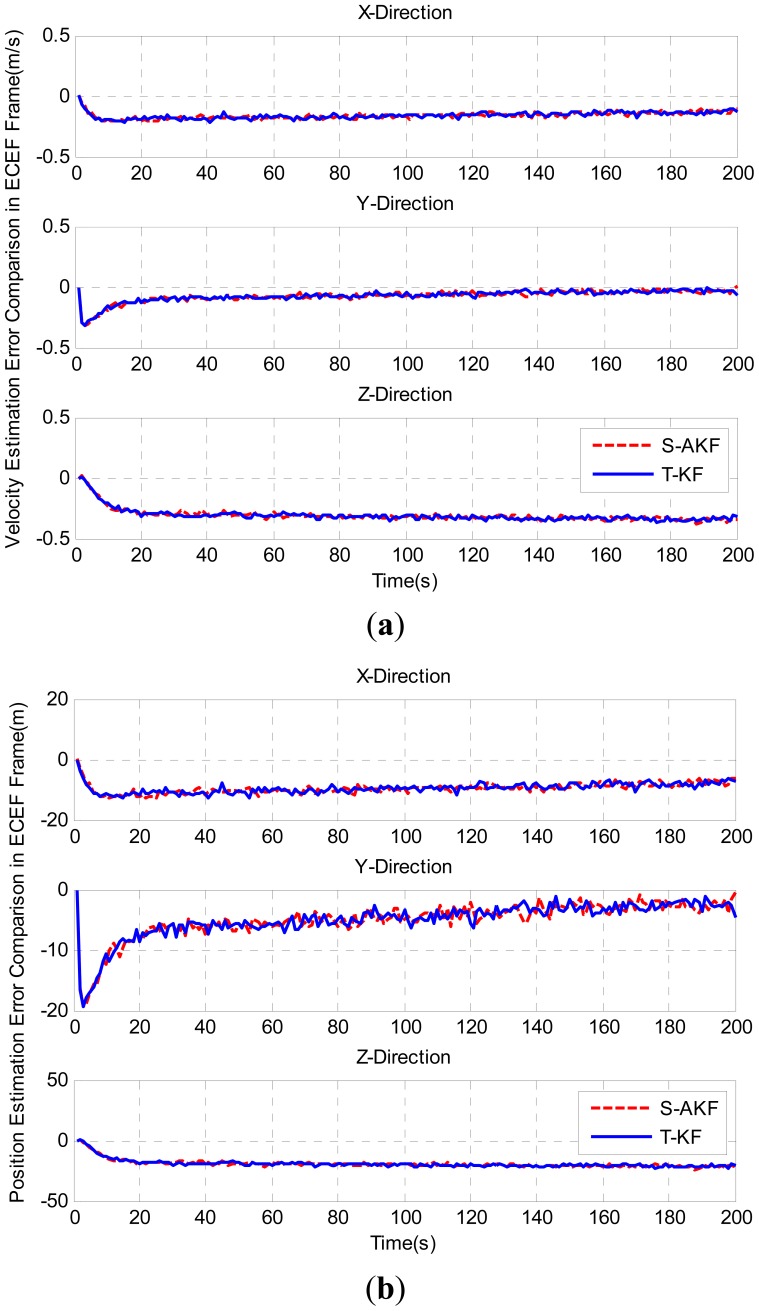
(**a**) Velocity estimation errors of S-AKF and T-KF in ECEF frame; (**b**) Velocity estimation errors of S-AKF and T-KF in ECEF frame.

For the field test case, the estimated velocity and position errors with S-AKF and T-KF in ECEF frame are shown in Figure 12(a,b), respectively. The statistics of position and velocity estimation errors are summarized in Table 6, where the S-AKF and T-KF showed almost similar velocity and position estimation errors.

## 5. Conclusions and Future Work

This paper investigated a simplified prefilter model for the ultratight COMPASS/INS integrated system. When compared to a traditional 5-dimension state prefilter model, the normalized signal amplitude and code tracking error were excluded, and only carrier phase, carrier frequency and carrier frequency rate tracking errors were included in the state space. However, as the code is not considered and there is a possibility of code/carrier divergence resulting in degradation in the carrier tracking process, an adaptive Kalman filter has been used to compensate for the divergence. Based on the COMPASS B3 frequency signal a federated COMPASS/INS integration system was implemented in software. A hardware sampling system was constructed to collect COMPASS IF data and INS data. Simulation and field tests showed an almost similar tracking and navigation performances of federated COMPASS/INS integration with traditional prefilter model and the proposed models. However, scalar measurement prefilter model with a 3-dimension state, even with an inclusion of an adaptive Kalman filter, has been observed to have significant reduction in the calculation load when compared to a traditional 5-dimension state and 2-dimension measurement prefilter model.

As with the GPS case, the ultra-tight COMPASS/INS integration shows an advantage over independent COMPASS receivers, particularly in low signal-to-noise and high dynamics environment. Only a static field test and a high dynamic simulation test were conducted for this analysis, and the future work will focus on further quantifying the benefits of ultra-tight COMPASS/INS integrations. The same simulation or field test will be conducted as the COMPASS, which is still a ‘local navigation’ satellite system, evolves into a global navigation satellite system in the future. Finally, although the COMPASS IF data and INS data collection was implemented in hardware, and the ultra-tight integration part was implemented in software, the future work will focus on the hardware implementation for the entire system.

## Figures and Tables

**Table 1.** Computational Complexities of Kalman filter implementation.

**Operation**	**Simplified prefilter model with adaptive Kalman filter**	**Traditional prefilter model**
	State dimension *n* = 3 Measurement dimension *l* = 1	State dimension *n* = 5 Measurement dimension *l* = 2
*PH^T^*(*R* + *HPH^T^*)^−1^	2(*n*^2^*l* + *nl*^2^) + 1 = 25	$2(n^{2}l+nl^{2})+l^{3}+\frac{1}{2}l^{2}+\frac{1}{2}l=35$
Φ*X*	*n*^2^ = 9	*n*^2^ = 25
Φ*P*Φ*^T^*	2*n*^3^ =54	2*n*^3^ = 250
*K*(*Z* − *HX*)	1+*nl*=4	*l*^3^ + *nl* = 18
(*I* − *KH*)*^P^*	*n*^3^ + *n*^2^*l*=36	*n*^3^ + *n*^2^*l* =175
$\overset{N}{\underset{m=1}{\text{∑}}}\Delta X_{m}\Delta X_{m}^{T}$	*n*^2^*N* = 9*N*	0
Total number of multiplications	128 + 9*N*	503

**Table 2.** Parameters defined in software-defined COMPASS Receiver.

**Parameter**	**Values**

Coherent integration time	1 ms
Prefilter update period	1 ms
Correlator spacing	0.5 chip
Adaptive window length (*N*)	5
*h*_0_	1.82 × 10^−21^ (*s*^2^/Hz)
*h*_−2_	1.51 × 10^−20^ (1/Hz)

**Table 3.** RMS Doppler frequency errors of tracked SVs with different prefilter models.

**Prefilter model for federated ultra-tight COMPASS/INS integration**	**RMS Doppler frequency estimation error per PRN (Hz)**	

	SV = 04	SV = 05

Simplified prefilter model with adaptive Kalman filter	3.773	3.094
Traditional prefilter model	3.687	2.974

**Table 4.** Statistics of position and velocity errors in ECEF frame with different prefilter models.

**Prefilter model for federated ultra-tight COMPASS/INS integration**	**Simplified prefilter model with adaptive Kalman filter**			**Traditional prefilter model**		

	X	Y	Z	X	Y	Z

Mean Position Error(m)	−13.436	−5.886	−30.279	−14.064	−6.036	−30.936
Std Position Error(m)	4.372	3.612	4.973	4.153	3.687	5.378
Mean Velocity Error(m/s)	−0.623	−0.267	−1.501	−0.639	−0.274	−1.406
Std Velocity Error(m/s)	0.199	0.207	0.237	0.188	0.197	0.244

**Table 5.** RMS Doppler frequency errors of tracked SVs with different prefilter models.

**Prefilter model for federated ultra-tight COMPASS/INS integration**	**RMS Doppler frequency estimation error per PRN (Hz)**	

	SV = 01	SV = 03

Simplified prefilter model with adaptive Kalman filter	0.663	0.744
Traditional prefilter model	0.613	0.778

**Table 6.** Statistics of position and velocity errors in ECEF frame with different prefilter models.

**Prefilter model for federated ultra-tight COMPASS/INS integration**	**Simplified prefilter model with adaptive Kalman filter**			**Traditional prefilter model**		

	X	Y	Z	X	Y	Z

Mean Position Error(m)	−9.231	−4.952	−18.953	−9.543	−5.011	−19.256
Std Position Error(m)	1.591	2.748	3.316	1.697	3.031	3.443
Mean Velocity Error(m/s)	−0.151	−0.069	−0.299	−0.164	−0.074	−0.312
Std Velocity Error(m/s)	0.030	0.047	0.052	0.028	0.049	0.055
